# Perivascular phosphorylated TDP‐43 inclusions are associated with Alzheimer's disease pathology and loss of CD146 and Aquaporin‐4

**DOI:** 10.1111/bpa.13304

**Published:** 2024-09-09

**Authors:** Jessica Santiago, Dovilė Pocevičiūtė, The Netherlands Brain Bank, Malin Wennström

**Affiliations:** ^1^ Cognitive Disorder Research Unit, Department of Clinical Sciences Malmö Lund University Malmö Sweden; ^2^ Netherlands Institute for Neuroscience Amsterdam The Netherlands

**Keywords:** astrocytic endfeet, BBB, glymphatic system, inclusions, MCAM, trans‐active response DNA binding protein of 43 kDa

## Abstract

The majority of patients with Alzheimer's disease (AD) exhibit aggregates of Trans‐active response DNA binding protein 43 (TDP‐43) in their hippocampus, which is associated with a more aggressive disease progression. The TDP‐43 inclusions are commonly found in neurons, but also in astrocytes. The impact of the inclusions in astrocytes is less known. In the current study, we investigate the presence of phosphorylated TDP‐43 (pTDP‐43) inclusions in astrocytic endfeet and their potential association with blood–brain barrier (BBB) damage, glymphatic system dysfunction, and AD pathology. By staining postmortem hippocampal sections from AD patients and non‐demented controls against TDP‐43 and pTDP‐43 together with the astrocytic markers glial fibrillary acidic protein (GFAP), astrocytic endfeet marker Aquaporin‐4 (AQP4), and markers for BBB alterations (CD146) and leakiness (Immunoglobulin A), we demonstrate a close association between perivascular pTDP‐43 or TDP‐43 inclusions and GFAP or AQP4. These perivascular inclusions were more prominent in AD and correlated with the disease severity and loss of CD146 and AQP4. The findings indicate a relationship between pTDP‐43 accumulation in astrocytic endfeet and BBB and glymphatic system dysfunction, which may contribute to the downstream pathological events seen in AD patients and the aggressive disease progression.

## Graphical abstract

**Hypothesis:** In Alzheimer's Disease (AD) patients, pTDP‐43 inclusions accumulate in astrocytic endfeet, leading to a loss of CD146 and Aquaporin‐4. This loss results in blood‐brain barrier breakdown and dysfunction of the glymphatic system, which may contribute to the aggressive disease progression seen in AD patients with TDP‐43 pathology. Created with BioRender.com.

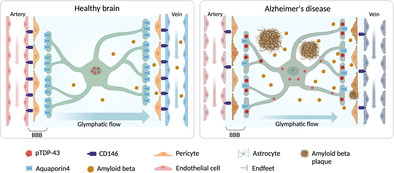

## 1 INTRODUCTION

Accumulation and deposition of amyloid‐beta (Aβ) is a key event in Alzheimer's disease (AD) thought to underly the neuronal loss and brain atrophy seen in these patients. The accumulation can be due to an increased Aβ production, as in the case of familial AD, but also to an impeded clearance of the peptide. Such dysfunction is thought to underly sporadic forms of AD and arises when systems involved in clearing excess Aβ are disrupted or altered. The blood–brain barrier (BBB), formed by pericytes, astrocytic endfeet, and endothelial cells, is one of these clearance systems known to be affected in AD. The impairment of the system is characterized by a reduced amount of endothelial‐pericyte tight junctions, loss of pericytes [35, 36], and increased infiltration of fibrinogen [23] and albumin from the periphery [18, 37, 45]. The glymphatic system is another system that is known to play an important role in Aβ clearance. Its function is dependent on the astrocytic endfeet which forms a fluid‐filled perivascular space around the brain vessels. The perivascular fluid is transported into the parenchyma via Aquaporin‐4 channels (AQP4) expressed on the astrocytic endfeet, and a bulk flow of interstitial fluid is created. This bulk flow pushes the fluid, along with unwanted soluble toxic substances such as Aβ and islet amyloid polypeptide, toward veins where it is taken up and transported away [33]. Patients with AD show both reduced expression of AQP4 and increased perivascular space [5], pointing toward a malfunctioning glymphatic system. Impairment of both clearing systems (i.e., BBB/pericytes and the glymphatic system) contributes to an accumulation of the amyloid peptides, which start to seed each other and deposit in the vessel walls and brain parenchyma [16].

Trans‐active response DNA binding protein of 43 kDa (TDP‐43) is a ubiquitous protein essential for the development of the central nervous system and known to regulate pathways that affect cell survival, metabolism, mitochondrial function, and synaptic function dependent on the tissue [1, 29]. The majority of TDP‐43 is nuclear during physiological conditions, with a small portion being shuttled between the nucleus and the cytoplasm. During stress conditions (such as oxidative stress) and inflammatory events [11], TDP‐43 is released to the cytoplasm where it regulates the formation of stress granules, ribonucleoprotein transport granules, translation, and other processes [32]. Under such pathological conditions, TDP‐43 can be hyperphosphorylated (pTDP‐43) and ubiquitinated [9], and despite being labeled for degradation, pTDP‐43 starts to accumulate in the cytoplasm in the form of inclusions. These inclusions are foremost associated with frontotemporal lobar degeneration (FTLD) and amyotrophic lateral sclerosis (ALS), as neuronal inclusions of the pTDP‐43 are a neuropathological hallmark of these diseases. However, in recent years it has become evident that more than 57% of all AD patients demonstrate hippocampal pTDP‐43 inclusions, and that the inclusions are associated with faster cognitive decline and a more aggressive disease course [27]. The pTDP‐43 inclusions have foremost been reported to occur in neurons, where they cause neurotoxicity and cytoskeletal dysfunction [13, 41], but the peptide can also aggregate and deposit in astrocytes [26, 40]. The consequences of the astrocytic pTDP‐43 inclusions are little known, although a few studies suggest that the neuroprotective properties of astrocytes are attenuated when pTDP‐43 accumulates and forms inclusions inside the cells [24, 25]. Interestingly, a study using electron microscopy has found small TDP‐43 inclusions associated with vessels in brain tissue from FTLD patients [26]. The authors of this article suggested that they were located inside the astrocytic endfeet. Although this finding needs to be verified, it raises the question of whether TDP‐43 inclusions affect the astrocytic endfeet function. Indeed, patients with FTLD show BBB permeability [18, 43] and inclusions of pTDP‐43 have been found in the vessel walls in the frontal cortex of ALS patients [14]. Moreover, mice overexpressing TDP‐43 show BBB permeability [43] and the loss of TDP‐43 function, which happens when TDP‐43 mislocates and aggregates, causes a reduction in blood circulation and vessel mispatterning in zebrafish [34]. Whether TDP‐43 has a direct implication on glymphatic failure is not yet determined, but a study on a TDP‐43 ALS mouse model showed that the glymphatic system is already impaired in the early stages of the disease [42]. In addition, TDP‐43 reduces the efficacy of AQP4 integration into the cell membrane [22]. The aim of the current study was to further investigate the presence of pTDP‐43 inclusions in astrocytic endfeet and determine if the accumulation of inclusions is associated with AD pathology, BBB damage, and alterations in the glymphatic system.

## 2 MATERIALS AND METHODS

### 2.1 Cases included in the study

The study was performed on hippocampi samples from (*n* = 16) non‐demented controls (NDC) and (*n* = 21) clinically and postmortem‐verified AD cases (The Netherlands Brain Bank (NBB)). The neuropathological assessment was performed according to Braak stages of neurofibrillary tangles (NFT) (stages I–VI) [7], the presence of Aβ plaques classified into four levels: O, A, B, and C (representing none, some, moderate amount, and many plaques, respectively) [7], and Lewy bodies/threads (LB) (Braak stages 0–6) [8]. Demographics of the cases are found in Table 1 and the individual data are found in Table S1. Written consent was obtained from all patients or their relatives for the use of tissue and clinical data in research in accordance with the international declaration of Helsinki and Europe's code of conduct for Brain Banking. The collection procedures were approved by the Medical Ethics Review Committee of VU Medical Centre in Amsterdam (The Netherlands) and the study was approved by the Swedish Ethical Review Authority.

**TABLE 1:** Cases included in the study.

	NDC (*n* = 16)	AD (*n* = 21)
Age (mean years ± SD)	77.4 ± 13.9	79.8 ± 10.8
Females (%)	68.8%	47.6%
*APOE4* (%)	18.8%	76.2%

Abbreviations: AD, Alzheimer's disease; *APOE4*, Apolipoprotein 4 carriers; NDC, non‐demented control.

### 2.2 Immunostainings

The hippocampi samples were either immersion‐fixed in 4% paraformaldehyde directly after autopsy (F1) or shortly after being snap frozen after autopsy (F2). The samples were then left in 30% sucrose for 3 days before they were sectioned into 40 μm and kept in an anti‐freezing solution at −20°C until they were used for immunostaining. Sections of all samples (F1 + F2) ((*n* = 16) NDC, (*n* = 21) AD) were co‐immunostained against pTDP‐43 and GFAP, while F1 sections ((*n* = 11) NDC, (*n* = 8) AD) were co‐stained against AQP4 and TDP‐43 and F2 sections ((*n* = 7) NDC, (*n* = 11) AD) were stained against CD146 (Figure S1). Sections from (*n* = 2) AD patient and (*n* = 2) NDC were also stained against GFAP and laminin (F1 + F2) and AQP4 and CD146 (F1). The primary antibodies used are specified in Table 2. After being washed in phosphate‐buffered saline containing potassium (KPBS), the sections were incubated for 1 h in a blocking solution containing 5% goat serum (Jackson Immunoresearch) and PBS with 0.25% Triton (KPBS+) at room temperature. The primary antibodies were then added to the blocking solution and left to incubate overnight at 4°C. The following day, the sections were washed and incubated for 2 h with the appropriate fluorochrome‐conjugated secondary antibody at room temperature. Biotinylated goat anti‐chicken antibody (VectorLaboratories) was used as a secondary antibody in the GFAP immunostaining, followed by incubation with Streptavidin 488 (VectorLaboratories) for 1 h. Lastly, all stained sections were incubated with Sudan Black (1% in 70% ethanol) (Sigma‐Aldrich) for 5 min, washed, and mounted with Vectashield Set mounting medium containing 4′,6‐diamidino‐2‐phenylindole (DAPI) (VectorLaboratories).

**TABLE 2:** Primary antibodies.

Antibody	Host	Dilution	Cat.no	Source
TDP‐43	Mouse	1:2500	60,019‐2‐Ig	Proteintech
pTDP‐43 (Ser 409/410)	Rabbit	1:400	80,007‐1‐RR	Proteintech
AQP4	Rabbit	1:1000	HPA014784	Sigma
GFAP	Chicken	1:1000	AB5541	Merck
CD146	Rabbit	1:600	HPA008848	Sigma
Laminin	Mouse	1:200	M0638	Dako

### 2.3 ImageJ area fraction analysis

To assess the area fraction of pTDP‐43 in the vessels, five images with three color channels were randomly captured from the molecular layer (ML) of cornu ammonis (CA1), of all subjects using a 40× objective in an OlympusAX70 light microscope equipped with Olympus DP72 camera. All images were analyzed using the Fiji software (ImageJ2 version 2.9.0). For the pTDP‐43, TDP‐43, AQP4, and CD146 quantification, five different *en face* vessels with an outer diameter between 10 and 15 μm (AQP4 and GFAP) and between 7–10 μm (CD146) were analyzed for each case. For the pTDP‐43 analysis, GFAP staining was used to manually delineate the vessel area using the polygon tool, while the AQP4 staining was used to delineate the vessel in the TDP‐43 staining. Sections stained against GFAP/laminin and AQP4/CD146 were used to define vessel‐associated GFAP and AQP4 as (i) enhanced staining (both AQP4 and GFAP) bordering a tubelike area in which the staining is absent and stained processes stretching toward the stained borders (GFAP), (ii) DAPI‐positive nuclei present within the tubelike area, which ensures that the absence of AQP4 or GFAP is not due to loss of tissue, and (iii) the contours of the tubelike area can be followed when moving in the Z plane. Representative images of AQP4 and GFAP associated with CD146 and laminin, respectively, are found in Figure S2. The selected area was added to the region of interest (ROI) manager and posteriorly applied to the pTDP‐43 or TDP‐43 channel to select the desired area of analysis. The inclusions inside the nuclei were excluded, which was done by applying an automated threshold to select the DAPI‐stained area, the selection was then added to the ROI manager and posteriorly applied to the pTDP‐43 channel to exclude nuclear inclusions from the analysis. Lastly, the same threshold value was applied to select the pTDP‐43 staining for all images, and the average size of pTDP‐43‐positive inclusions and the percentage of pTDP‐43 fluorescence per total area were acquired using the measure tool. For the AQP4 and CD146 analysis, the AQP4 and CD146 stainings, respectively, were used to delineate the vessel area. The total area fraction of the AQP4 or CD146 staining within the selected area was measured and divided by the total delineated area of the vessel. The area fraction of IgA from a subset of cases ((*n* = 5) NDC, (*n* = 9) AD) was analyzed in a previous study (for methods and materials see [31]). Associations between TDP‐43 or pTDP‐43 and GFAP or AQP4 were analyzed using confocal microscopy (Zeiss LCM 800).

### 2.4 Scoring of pTDP‐43 inclusions in the CA1 region

The presence of pTDP‐43 inclusions in the pyrimadal layer of CA1 of all cases' hippocampi was evaluated using a 0–5 scale, where 0 = no, 1 = very few, 2 = few, 3 = moderate, 4 = many, and 5 = very many of stained pTDP‐43 inclusions (CA1 pTDP‐43).

### 2.5 Statistical analyses

The statistical analyses were conducted using the SPSS version 28 (IBM Corp) software. Comparisons were performed using the Mann–Whitney U test. Correlations between the investigated variables were performed using the two‐tailed Spearman's correlation test. All correlations were performed with all the subjects in the groups NDC and AD, unless stated otherwise. Since there was a negative correlation between AQP4 area fraction and age in the NDC group, analysis of covariance (ANCOVA) was employed when comparing the difference between AD and NDC, while partial correlations (with age as a covariate) were used to investigate the correlations with other variables. The values of pTDP‐43 were subjected to logarithmic transformation before creating comparative graphs and correlation scatter plots, but the analysis was performed on original values using the Mann–Whitney *U* test and Spearman's correlation test. Correlations and differences were considered significant at *p* ≤ 0.05.

## 3 RESULTS

### 3.1 pTDP‐43 inclusions are found in astrocytes

**FIGURE 1:**
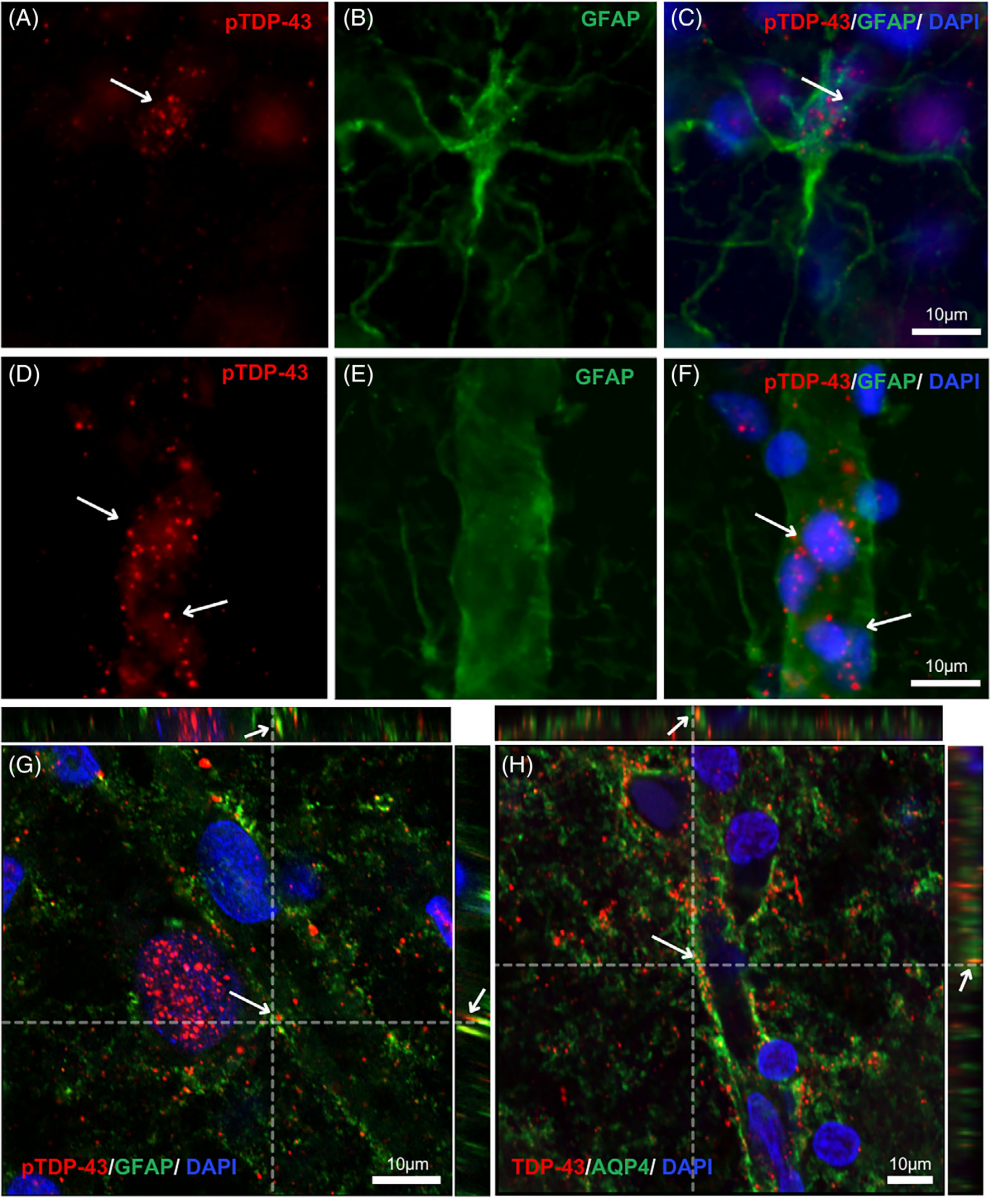
Co‐immunofluorescent staining against pTDP‐43 or TDP‐43 (red), GFAP or AQP4 (green), and DAPI (blue) of the ML of CA1 area of the hippocampus of a case with AD. Inclusions of pTDP‐43 were found in the cell bodies of astrocytes (A–C) and in the near vicinity of GFAP associated with small vessels (D–F). Fluorescent immunohistochemistry and z‐stack analysis with confocal microscopy revealed that pTDP‐43‐stained inclusions (red, indicated with arrows) are encountered in close proximity to GFAP‐stained structures (green) (G). In (H), TDP‐43‐stained inclusions (red, indicated with arrows) are surrounded by AQP4‐stained regions (green). Dashed lines in G and H indicate the position of the z‐stack images.

To examine the presence and localization of pTPD‐43 in astrocytes, we immunostained AD sections against pTDP‐43 and the astrocytic marker GFAP. The staining showed that pTDP‐43 inclusions could be found both within GFAP‐positive astrocytic cell bodies (Figure 1A–C) and in close vicinity to GFAP associated with small vessels (Figure 1D–F). The close association between pTDP‐43 inclusions and vessel‐associated GFAP was further confirmed by confocal microscopy (Figure 1G). Since GFAP is an intermediate filament, staining against the marker is not sufficient to determine whether the vessel/GFAP‐associated inclusions are localized inside astrocytic endfeet. Therefore, we also stained sections against AQP4, which is found in the astrocytic endfeet membrane. Due to a shortage of reliable AQP4 antibodies made in a host different from our pTDP‐43 antibody (rabbit), we chose to instead co‐stain AQP4 together with mouse monoclonal TDP‐43 antibody. The TDP‐43 staining again revealed TDP‐43 inclusions in the vicinity of vessels and confocal microscopy showed that these TDP‐43‐positive inclusions were surrounded by AQP4‐stained regions (Figure 1H).

### 3.2 The load of perivascular pTDP‐43 inclusions increases in Alzheimer's disease patients

**FIGURE 2:**
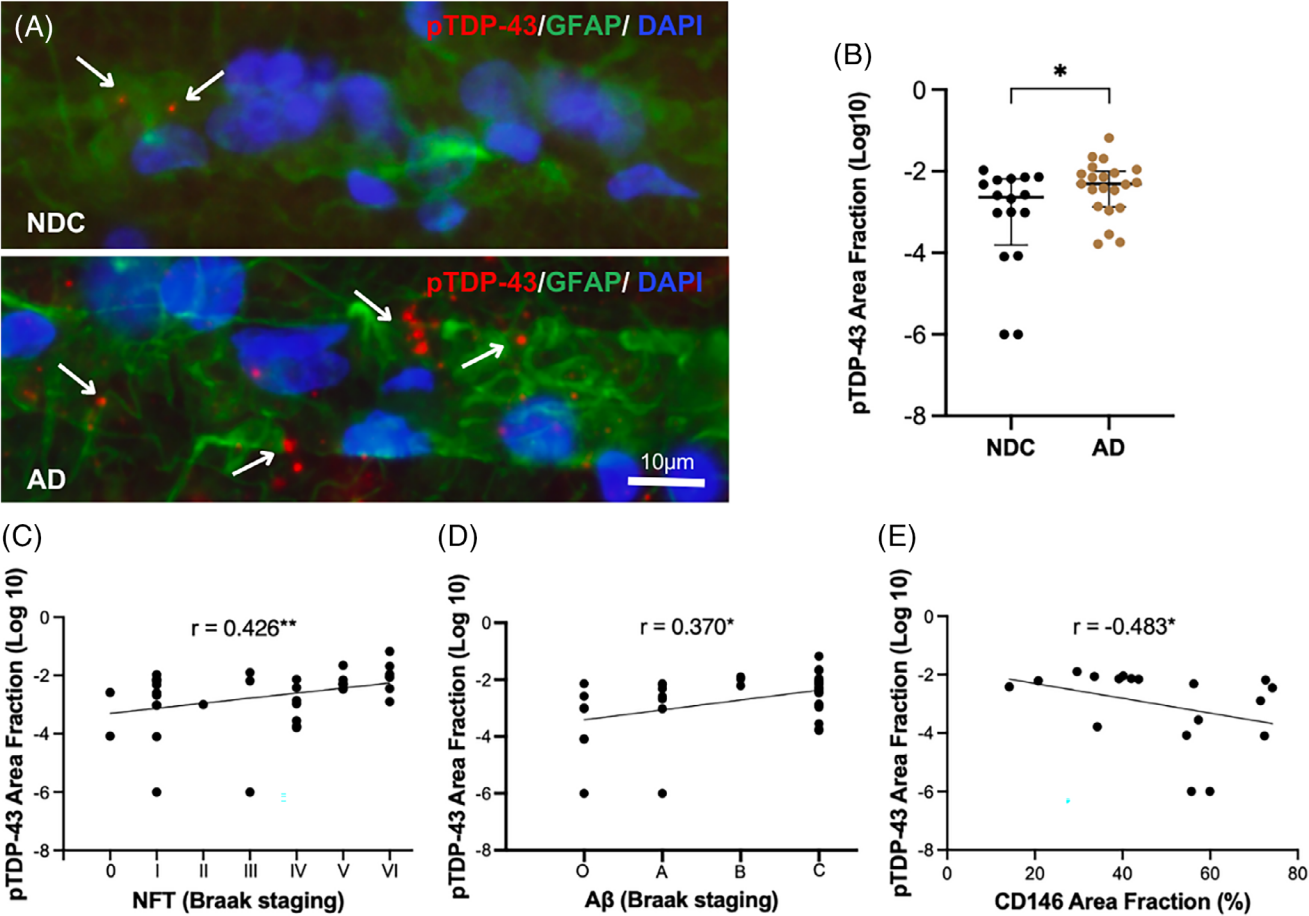
Immunostainings of NDC and AD brains indicate an increased number and size of GFAP‐associated pTDP‐43 inclusions (A). ImageJ analysis confirms a significantly higher pTDP‐43 area fraction in AD compared to NDC (B). The area fraction of perivascular pTDP‐43 inclusions correlated positively with the NFT (C) and Aβ plaque Braak scores (D). There was also a negative correlation between pTDP‐43 and CD146 area fractions around small vessels (E). Data in (B) were analyzed using the Mann–Whitney *U* test. The correlations in (C,D) were analyzed using Spearman's and in (E) Pearson's correlation tests. Each point represents a mean of the analysis of five different small vessels from each case. *p < 0.05, **p < 0.01.

Next, we analyzed the load of perivascular inclusions of pTDP‐43 in the ML of CA1 region of AD patients and NDC. Our analysis showed a significantly higher pTDP‐43 area fraction/vessel area in AD patients compared to NDC (*p* = 0.047) (Figure 2A,B and Figure S3). The pTDP‐43 values did not correlate with age and no significant differences between *APOE4* carriers and noncarriers as well as between females and males were found. The area fraction of pTDP‐43 correlated positively with NFT Braak stages (*p* = 0.009) and Aβ Braak stages (*p* = 0.024) (Figure 2C,D), but not with LB stages (*r* = −0.157, *p* = 0.352). The correlation between pTDP‐43 and NFT was still significant within the AD group (*r* = 0.450, *p* = 0.041). To investigate if the perivascular pTDP‐43 inclusions correlate with the pTDP‐43 pathology commonly seen in the CA regions of AD patients [17], we also scored the presence of pTDP‐43 inclusions in the pyramidal region of CA1 (CA1 pTDP‐43). Although we found significantly higher scores of CA1 pTDP‐43 inclusions in AD patients compared to NDC (*p* < 0.001, Figure S3), the scores did not correlate significantly with the perivascular pTDP‐43 inclusions (*r* = 0.280, *p* = 0.098). They did, however, correlate positively with both NFT Braak stages (*p* = 0.008) and Aβ Braak stages (*p* < 0.001) (Figure S3), but not with LB stages (*r* = −0.178, *p* = 0.298).

### 3.3 Perivascular pTDP‐43 inclusions are associated with a reduction in CD146 expression

To investigate if the increased presence of perivascular pTDP‐43 inclusions in the ML of CA1 is associated with alterations in the BBB integrity, we analyzed the correlations between perivascular pTDP‐43 area fraction and area fractions of IgA and CD146 of AD patients and NDC. Infiltration of the former is linked to BBB leakiness, while the latter is an adhesion molecule shown to mediate the interaction between endothelial cells and pericytes and thereby promote BBB formation [10]. No significant correlation was found between IgA and perivascular pTDP‐43 area fractions (*r* = 0.196, *p* = 0.503), but the area fraction of pTDP‐43 correlated negatively with the area fraction of CD146 (*p* = 0.042) (Figure 2E). The CD146 area fraction did not differ between NDC and AD (*p* = 0.425), but as reported previously [31], the IgA area fraction was higher in AD compared to NDC (*p* = 0.042) (Figure S4). No correlations were found between CA1 pTDP‐43 scores and IgA (*r* = 0.339, *p* = 0.258) or CD146 values (*r* = −0.368, *p* = 0.146).

### 3.4 Decreased AQP4 expression in AD patients is associated with pTDP‐43 inclusions

**FIGURE 3:**
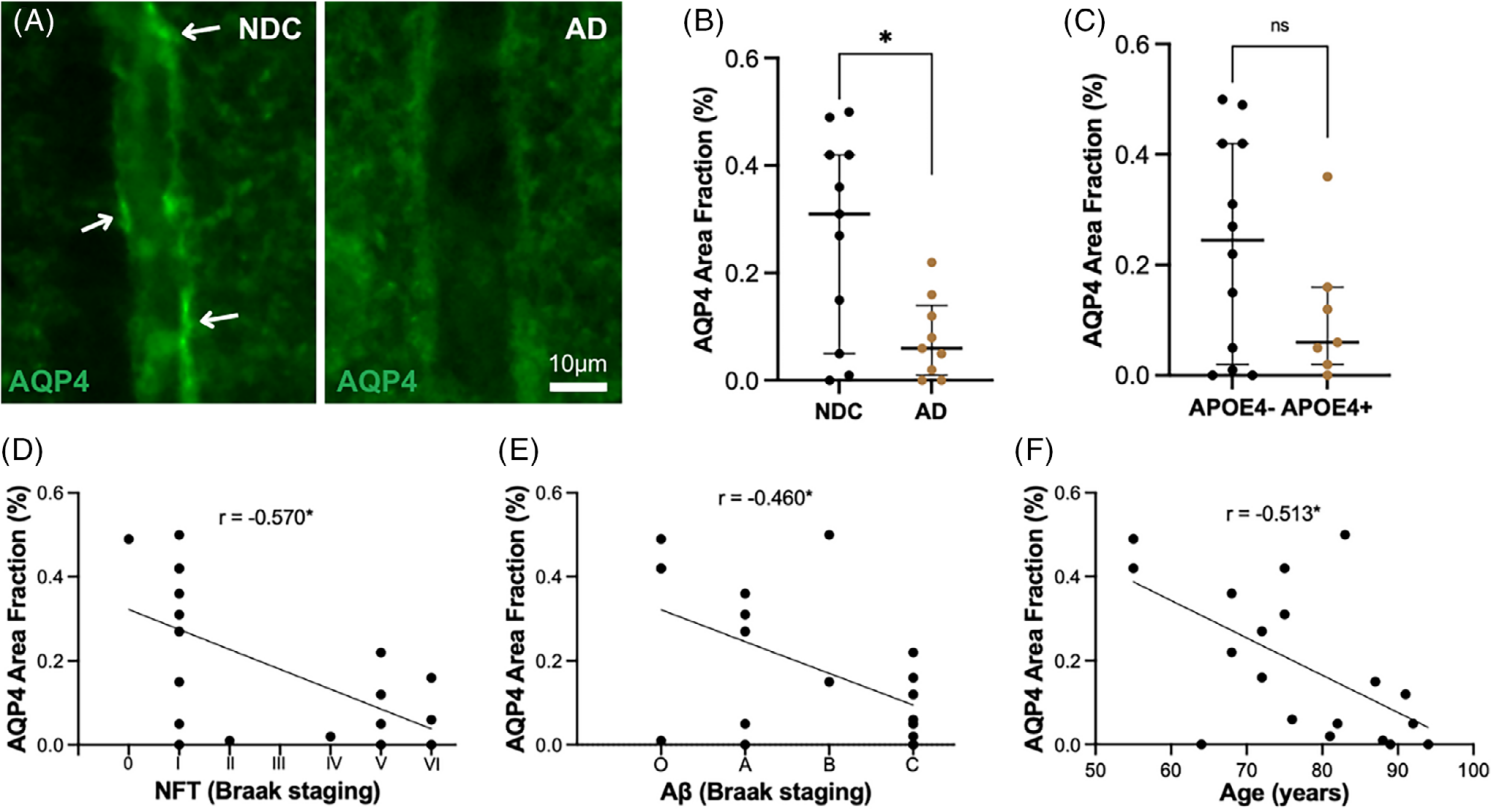
Immunofluorescent staining against AQP4 suggested a lower concentration of AQP4 on the walls of small vessels in AD brains compared to NDC (A). ImageJ analysis confirmed a significantly lower AQP4 area fraction in small vessels of AD cases compared to NDC (B) and a trend toward lower AQP4 area fraction in *APOE4*‐positive compared to *APOE4*‐negative groups (C). The stained area of AQP4 in small vessels was also negatively associated with NFT Braak staging (D), Aβ plaque Braak scores (E), and age (F). Data in (B and C) were analyzed using the Student's *t*‐test, in (D and E) using Spearman, and in (F) Pearson correlations tests. Each point represents a mean of the analysis of five different vessels from each case. *p < 0.05, **p < 0.01.

Finally, we investigated if the increased presence of perivascular TDP‐43 and pTDP‐43 inclusions in the ML of CA1 is associated with alterations in the AQP4 expressed by astrocytic endfeet. Examination of the AQP4‐stained area fraction indicated that cases with AD had a lower AQP4‐stained area fraction compared to NDC (*p* = 0.041) (Figure 3A,B). No difference between *APOE4* noncarriers and carriers was detected (0.25 (0.02, 0.42) vs. 0.63 (0.02, 0.15), *p* = 0.261) (Figure 3C). The AQP4 area fraction correlated negatively with NFT (*p* = 0.011) and Aβ plaque (*p* = 0.048) Braak scores (Figure 3D,E). The area fraction of perivascular AQP4 did not correlate with TDP‐43 (*r* = −0.116, *p* = 0.637), pTDP‐43 (*r* = −0.130, *p* = 0.596), or CA1 pTDP‐43 (*r* = −0.249, *p* = 0.412). However, we noted a correlation between the area fraction of AQP4 and age (*r* = −0.596, *p* = 0.025) (Figure 3F). This correlation remained when only the NDC group was analyzed (*r* = −0.690, *p* = 0.011). Therefore, we also analyzed the values using age as a covariate. The significant difference in the area fraction of AQP4 between NDC and AD remained after correcting for age (*p* = 0.007). Furthermore, *APOE4* carriers showed a lowered area fraction of AQP4 compared to noncarriers (*p* = 0.044). Also, the negative correlations between AQP4 and NFT and Aβ plaque scores remained significant (*r* = −0.658, *p* = 0.003 and *r* = −0.471, *p* = 0.048, respectively). After correcting for age, perivascular AQP4 correlated significantly with pTDP‐43 perivascular inclusions (*r* = −0.537, *p* = 0.021), but not with TDP‐43 (*r* = −0.088, *p* = 0.727) or CA1 pTDP‐43 (*r* = −0.057, *p* = 0.822). There was no statistically significant difference in AQP4 area fraction between males and females before or after including age as a covariate (0.11 (0.014, 0.31) vs. 0.15 (0.02, 0.41), *p* = 0.657; *p* = 0.420 after correction).

## 4 DISCUSSION

Our study demonstrates the presence of pTDP‐43 inclusions in astrocytic endfeet in the CA1 region of AD patients. The study also shows an association between the load of these inclusions and AD pathology and disease severity. Our findings further link the accumulation of pTDP‐43 inclusions to alterations in BBB integrity and reduction in astrocytic AQP4 expression.

The presence of pTDP‐43 inclusions in astrocytes aligns with previous research documenting similar findings in other neurodegenerative disorders like FTLD and ALS [2, 12, 14, 28]. Our study also confirmed the presence of TDP‐43 specifically within astrocytic endfeet, as suggested by a previous study investigating microvasculopathy in FTLD using immunoelectron microscopy [26]. The perivascular pTDP‐43 inclusions were more pronounced in AD patients. Whether this is a result of increased pTDP‐43 accumulation due to the AD pathology or if the inclusions themselves induce the AD pathology is yet to be determined, but a previous mouse study demonstrating an exacerbation of AD pathology in animals injected with TDP‐43 [38] is in favor to the latter hypothesis. It is further interesting that the inclusion load correlated positively with NFT Braak stages and Aβ burden. Although the specific role of perivascular TDP‐43 inclusions in AD has not been studied before, it is noteworthy that our findings support the previous epidemiological research indicating a worse prognosis in AD patients with TDP‐43 inclusions [19, 20] and patients with quadruple misfolded proteins (Aβ, tau, alpha‐synuclein, and TDP‐43) [21]. However, it should be noted that our study failed to find a correlation between perivascular pTDP‐43 and pTDP‐43 in the pyrimadal region of CA1, even though the pTDP‐43 inclusion load in this region is also increased in AD patients. Such an increase has been reported before and the pTDP‐43 inclusions were primarily found in the pyramidal neurons of the hippocampus [3, 15]. It may thus be that neuronal accumulation of pTDP‐43 is only partly associated with the astrocytic perivascular accumulation of pTDP‐43 and involves slightly different pathological events.

Our results also demonstrated an inverse relationship between perivascular pTDP‐43 and alterations in CD146 expression. This adhesion molecule is located at the intercellular junctions of endothelial cells and promotes BBB formation by mediating the interaction between endothelial cells and pericytes [4]. During neuroinflammatory conditions, CD146 is shed from the cell surface, and elevated levels of soluble CD146 in cerebrospinal fluid are associated with BBB damage [39]. The link between TDP‐43 pathology and BBB alterations has been shown before. Patients with FTLD show increased BBB permeability [18, 43] and loss of mural cells (which include BBB‐forming pericytes) has been found to correlate with TDP‐43 pathology in the cortex of AD patients [6]. Preclinical studies further show that overexpression of TDP‐43 in mice causes BBB permeability [43] and vessels in TDP‐43 zebrafish models appear distorted [34]. These findings collectively indicate an association of TDP‐43 pathology with BBB alterations. Of note, our studies did not find a correlation between IgA area fraction and pTDP‐43 inclusions, which suggests that pTDP‐43 is not associated with major BBB damage such as leakiness, but rather minor alterations in the BBB integrity. Additionally, although our IgA analysis, in line with previous studies [18, 37, 45], suggests changes in the BBB integrity in AD patients, the slight decrease in CD146 expression in AD patients did not reach significance when compared to NDC. The lack of detectable changes in the CD146 expression may be due to a lack of power and the fact that ImageJ is a rather crude analysis method but could also imply that changes in the CD146 expression are not an AD specific event. We also found a reduced presence of AQP4 around small vessels of AD patients compared to NDC along with a negative correlation with age and the severity of AD pathology both before and after correcting for age. These findings are in line with a previous study which also found a reduction in AQP4 in the frontal cortex of AD patients and negative correlations between AQP4 expression and NFT Braak stages as well as Aβ burden after correcting for age [44]. Given that APQ4 plays a crucial role in the glymphatic system, and thereby clearance of excessive extracellular Aβ and p‐tau, it is tempting to speculate that the glymphatic system is malfunctioning in AD patients and that this underlies the formation of tangles and accumulation of Aβ in the brain of these patients. The loss of AQP4 in AD may be due to several pathological events, but it is interesting that we found a negative correlation between perivascular pTDP‐43 and AQP4 area fractions after correcting for age. The association between AQP4 expression and age has been described before, but most studies have found a positive relationship [30, 44], rather than a negative, between normal aging and AQP4 expression. However, the positive correlations with age found in the former studies were with the overall AQP4 expression, not specifically the perivascular AQP4, in the analyzed brain regions of non‐demented individuals. However, one of the studies also analyzed the perivascular AQP4 and found no significant correlation with age, but, interestingly, the trend of the correlation was negative (*r* = −0.210, *p* = 0.150) [43]. In view of these previous findings and our own results, we speculate that AQP4 relocates from the astrocytic endfeet to other compartments of the cell, leaving the glymphatic system affected also in the aged brain.

It should further be noted that a correlation was not found between TDP‐43 and AQP4, which suggests that the AQP4 downregulation may be foremost related to inclusions of the TDP‐43 phosphorylated at Ser409/410. Although the relationship between AQP4 and pTDP‐43 needs to be investigated further, it is tempting to speculate that pTDP‐43 pathology may contribute to the AQP4 loss in AD patients. This idea is reinforced by a previous cell culture study showing a correlation between the depletion of nuclear TDP‐43 and the reduction of AQP4 localized on the endfeet membranes [22]. Additionally, studies on a TDP‐43 ALS mouse model show that the glymphatic system is impaired already in the early stages of the disease [42].

Lastly, the limitation of this study foremost concerns the relatively small sample sizes in different groups, which may increase the likelihood of false negatives and/or loss of potential relationships. Therefore, additional studies with larger cohorts are necessary to enhance the validity and generalizability of our findings. Another limitation concerns the limited neuropathological evaluation information. The brain tissue from the cases included in this study was collected and evaluated before 2014 and hence the TDP‐43 staging analysis has not been performed. Instead, the cases were neuropathologically evaluated using Braak stages for both tau and Aβ pathology, which at the time was practiced at the NBB. Hence, we have neither CERAD nor Thal staging values, which would be of interest to further explore the associations between perivascular pTDP‐43 pathology and other amyloid peptide aggregates. Finally, since our study is primarily observational, we encourage future experimental studies to unravel the temporal aspect of TDP‐43 accumulation, AD pathology, and alterations in the BBB and the glymphatic system.

## 5 CONCLUSION

Our study suggests that pTDP‐43 inclusions in the astrocytic endfeet can play a role in AD pathology progression by affecting brain clearance functions such as BBB integrity and the glymphatic system. Future studies investigating the direct impact of pTDP‐43 on AQP4 regulation and BBB are warranted as they might shed light on mechanisms implicated in the unfavorable prognosis of AD patients with TDP‐43 inclusions and point out possible therapeutic targets.

## AUTHOR CONTRIBUTIONS

J.S. and M.W. contributed to the study concept and design. J.S. performed the immunostainings and analyzed the data with support from D.P. and was a major contributor in writing the manuscript. D.P. performed and analyzed the IgA staining. The NBB contributed to the brain tissue and neuropathological evaluation. All authors have read and revised the manuscript for intellectual content and agreed to the final version of the manuscript.

## FUNDING INFORMATION

The study was funded by the Brain Foundation (FO2023‐0113), Crafoord Foundation (20230519), Olle Engkvists Foundation (227‐0209), Dementia Foundation (2023), Greta and Johan Kockska Foundation (2022), and the Åhlén Foundation (233002). None of the funders have been involved in the design of the study and collection, analysis, interpretation of data, and/or in the writing of the manuscript.

## CONFLICT OF INTEREST STATEMENT

The authors declare that they have no competing interests.

## ETHICS STATEMENT

Informed consent for using brain tissue as well as clinical data for research purposes was obtained from the patients or from their closest relatives in accordance with the International Declaration of Helsinki and the Code of Conduct for Brain Banking. The tissue collection protocols were approved by the medical ethics committee of VU Amsterdam and the Swedish Ethical Review Authority approved the study (Dnr 2021/04270). All data were analyzed anonymously.

## Supporting information


**Data S1.** Supporting Information.

## Data Availability

All data generated or analyzed during this study are included in this published article.
