# Supplementary material for: Perivascular phosphorylated TDP‐43 inclusions are associated with Alzheimer's disease pathology and loss of CD146 and Aquaporin‐4
Source: Brain Pathol. 2024 Sep 9;35(2):e13304. doi: 10.1111/bpa.13304 (PMC11835440; doi:10.1111/bpa.13304)
Supplement: Supplementary file 1 — Data S1. Supporting Information. [file BPA-35-e13304-s001.pdf]

Supplement Table S1. Table presenting the individual demographic data and the neuropathological evaluation of the cases included in the study.

Diagnosis	Age (years)	Biological sex	NFT (Braak)	Aβ (Braak)	LB (Braak)	*APOE* alleles	PMD (h:m)	Postfixation method
NDC[a, b]	102	male	III	A	0	43	05:00	F2
NDC[a, c]	55	male	0	O	0	33	07:30	F1
NDC[a, c]	55	female	I	O	0	33	07:30	F1
NDC[a, b]	60	female	0	O	0	32	08:10	F2
NDC[a, c]	68	male	I	A	0	43	06:20	F1
NDC[a, b]	70	male	I	O	3	32	06:20	F2
NDC[a, c]	72.	female	I	A	0	33	06:50	F1
NDC[a, b, c]	75	female	I	O	0	33	09:10	F1
NDC[a, b]	75	male	I	A	0	33	05:25	F2
NDC[a, c]	75	female	I	A	0	32	13:00	F1
NDC[a, b, c]	83	female	I	B	0	32	04:40	F1
NDC[a, b, c]	87	female	I	B	0	33	07:00	F1
NDC[a, c]	88	female	II	O	0	33	05:50	F1
NDC[a,c]	89	female	I	A	0	33	07:10	F1
NDC[a, b]	92	female	III	O	0	34	06:35	F2
NDC[a, c]	92	female	I	A	1	33	07:45	F1
AD[a, b]	63	male	IV	C	6	44	04:55	F2
AD[a]	64	female	IV	C	6	44	08:15	F1
AD[a, c]	64	male	VI	C	0	34	08:05	F1
AD[a, b]	65	female	V	C	0	43	09:15	F2
AD[a, c]	68	male	V	C	0	33	09:15	F1
AD[a]	69	male	VI	C	0	43	06:30	F2
AD[a, b]	70	female	VI	C	0	44	04:20	F2
AD[a, b]	71	female	V	C	0	43	04:15	F2
AD[a, c]	72	male	VI	C	0	43	05:15	F1
AD[a]	74	male	IV	C	6	34	05:15	F1
AD[a, c]	76	male	VI	C	0	44	05:10	F1
AD[a, b]	78	female	VI	C	0	44	04:45	F2
AD[a, c]	81	female	IV	C	0	43	08:10	F1
AD[a, c]	82	male	V	C	0	43	04:25	F1
AD[a, b]	83	male	III	B	0	43	06:40	F2
AD[a, b]	85	male	IV	C	0	33	08:35	F2
AD[a, b]	87	female	IV	C	0	33	06:40	F2
AD[a, b]	91	female	IV	C	0	33	06:40	F2
AD[a, c]	91	female	V	C	5	34	05:20	F1
AD[a, b]	92	female	VI	C	0	43	06:10	F2
AD[a, c]	94	male	V	C	0	33	04:15	F1

NDC=non-demented controls, AD=Alzheimers disease, NFT = neurofibrillary tangles, Aβ = amyloid beta, LB = Lewy bodies, *APOE* = apolipoprotein E, [a] = stained against pTDP-43, [b] = stained against CD146, and [c]= stained against AQP4. F1= Immersionfixed in formaldehyd directly at autopsy, F2= Immersionfixed in formaldehyde after snapfreezing at autopsy.

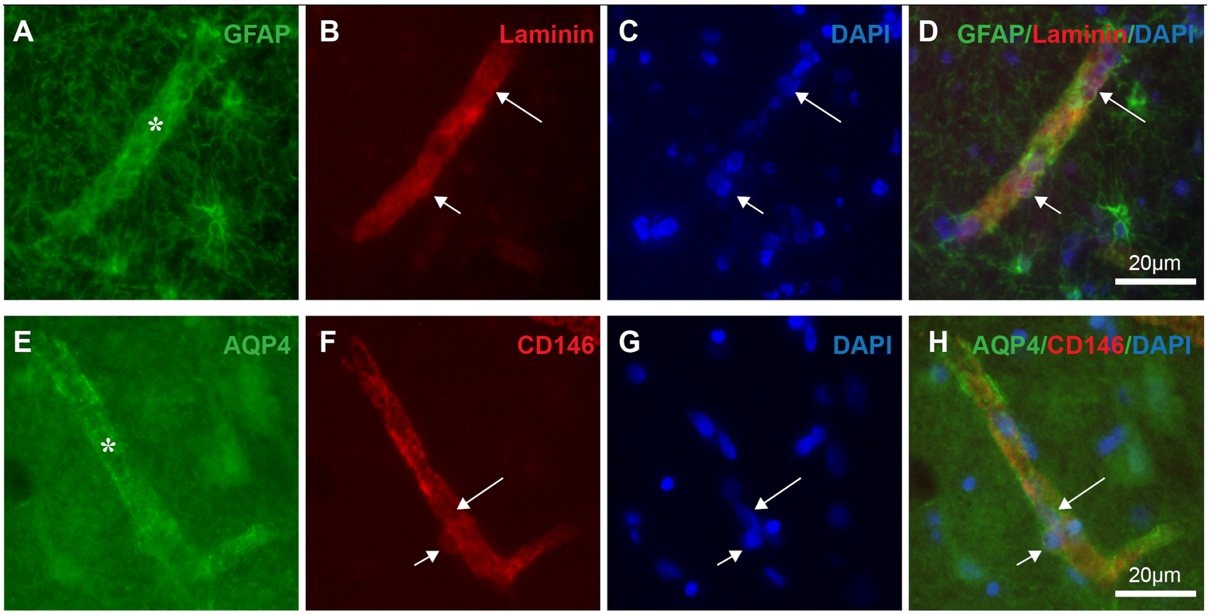

Supplement Figure S2. Representative images of immunostainings against glial fibrillaryacidic protein (GFAP) and Aquaporin4 (AQP4) in the molecular layer of CA1 fulfilling the definition criteria of vessel-associated GFAP and AQP4. Image in (A) shows GFAP positive processes (in green) stretching towards the vessel. The GFAP immunoreactivity is stronger (in green, indicated with asterix) where it encloses the laminin-embedded vessel (red in B and D). DAPI positive nuclei (blue in (C and D)) are found inside the tubelike GFAP formation and are enclosed by laminin, suggesting that they are either pericytes (indicate with a short arrow in B-D) or endothelial cells (indicated with a long arrow in B-D). Image in (E) shows AQP4 immunoreactivity (in green, indicated with asterix) bordering a vessel stained against a CD146 (red in F). DAPI positive nuclei are found inside the tubelike AQP4 formation and are enclosed by CD146 suggesting that they are either pericytes (indicated with a short arrow in F-H) or endothelial cells (indicated with a long arrow in F-H).

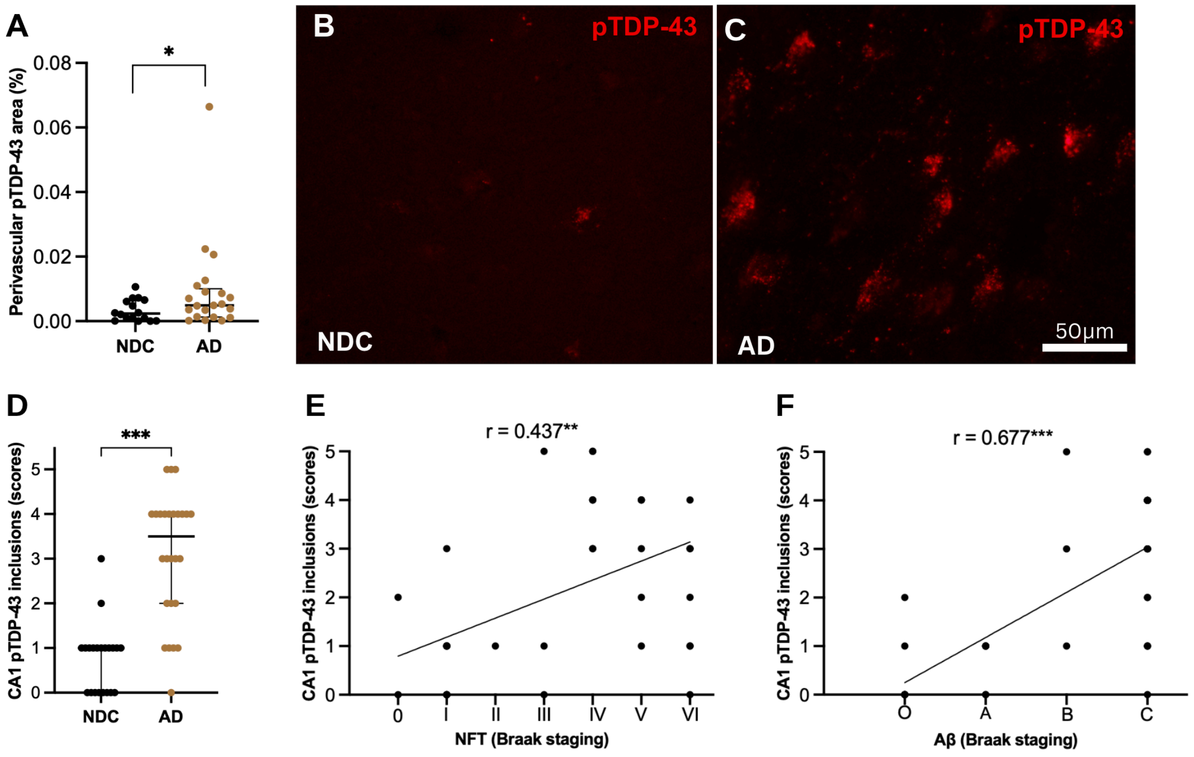

Supplement Figure S3. Graphs and images demonstrating perivascular pTDP-43 inclusions and pTDP-43 inclusions in the cornu ammonis (CA) 1 region. The graph in (A) shows the distribution of perivascular pTDP-43 before the logarithmic transformation. Images in (B and C) show pTDP-43 in the CA1 regions of a non-demented control (NDC) (B) and an Alzheimer's disease (AD) case (C). Graph in (D) shows the significant increase in pTDP-43 inclusions in the CA1 region (CA1 pTDP-43). Scatter plots in (E and F) demonstrate the correlations between CA1 pTDP-43 and neurofibrillary tangles (NFT) Braak stages (E) and amyloid beta (Aβ) Braak stages. Data in (A) and (D) are presented as median values with interquartile ranges and are analyzed using the Mann Whitney U-test. Correlations analysis (E and F) were performed using the Spearman's test. * = $p < 0.05$, ** = $p < 0.01$,*** = $p < 0.001$.

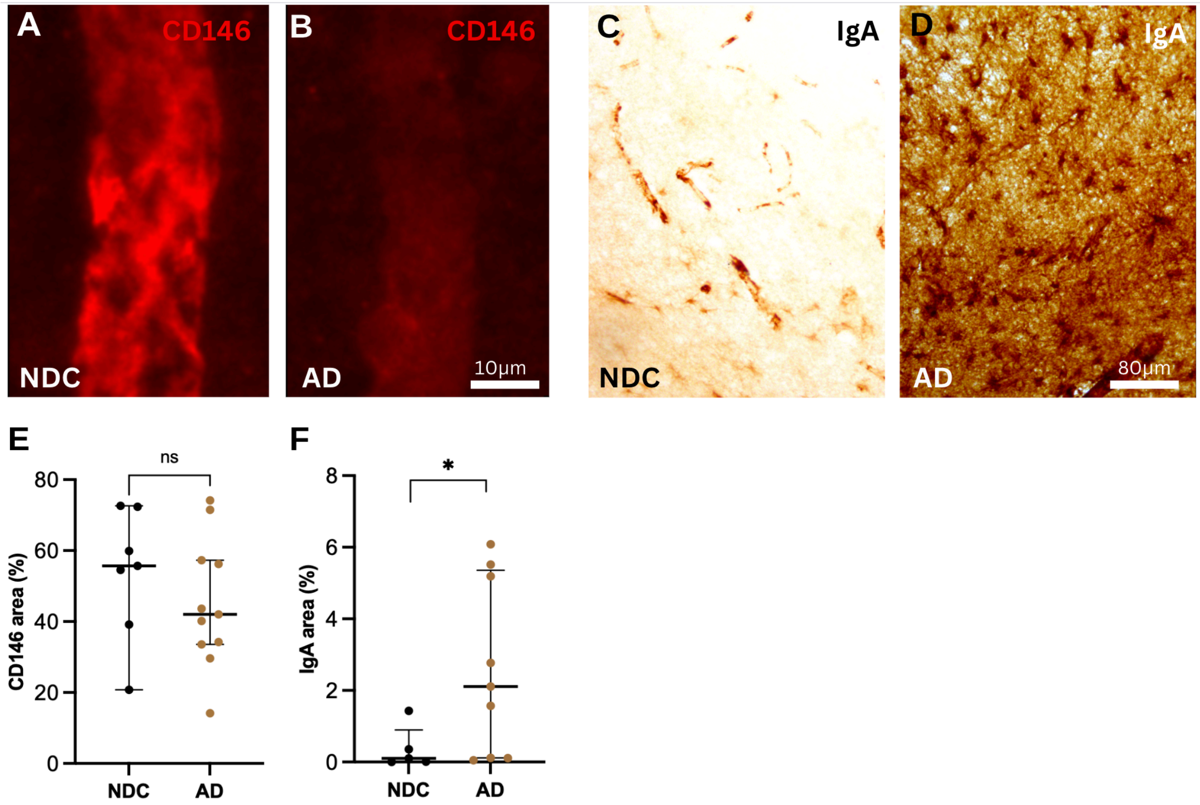

Supplement Figure S4. Representative images of immunostainings against CD146 and IgA in the stratum lacunosum-moleculare (SLM) and distribution of the CD146 and IgA area fraction values. Image in (A) shows the CD146 immunoreactivity in a non-demented control (NDC) with low presence of perivascular pTDP-43 inclusions, while image in (B) shows the CD146 immunoreactivity in an Alzheimer's disease (AD) patient with high presence of perivascular pTDP-43 inclusions. The same cases were immunostained for IgA and image in (C) shows the IgA immunoreactivity in the NDC with low presence of perivascular pTDP-43 inclusions and (D) shows the AD case with a high presence of perivascular pTDP-43 inclusions. Graphs in (E-F) show the distribution of CD146 (E) and IgA (F) and the significant difference between NDC and AD cases (F). Data in (E) and (F) are presented as median values with interquartile ranges and are analysed using the Mann Whitney U-test. *=$p<0.05$
